# The effect of resiquimod (a Toll-like receptor-7/8 agonist) on the balance between pro-inflammatory and anti-inflammatory cytokines in breast cancer patients

**DOI:** 10.1007/s12672-026-04619-8

**Published:** 2026-03-07

**Authors:** Eman ElAlfy, Lobna A. Abou-Shamaa, Soheir Rizk Demian, Eman M Osman, Yasmine ELwany, Marwa Mohamed Samak

**Affiliations:** 1 Cancer Management and Research Department, Medical Research Institute, Alexandria University, Alexandria, Egypt; 2 Immunology and Allergy Department, Medical Research Institute, Alexandria University, Alexandria, Egypt

**Keywords:** Breast cancer, Inflammation, Resiquimod, TLR agonist, Cytokines, IL-12, IL-35

## Abstract

**Background:**

Breast cancer (BC) progression is influenced by both pro- and anti-inflammatory cytokines. Interleukin (IL)-12 and IL-35 play opposing immunological roles, functioning as pro- and anti-inflammatory cytokines with anti- and pro-tumorigenic effects, respectively. Resiquimod (RSQ), a Toll-like receptor 7/8 (TLR7/8) agonist, is an immunomodulatory agent that enhances antitumor immune responses. However, the influence of RSQ on the balance between pro- and anti-inflammatory cytokines in BC patients remains poorly understood.

**Aim:**

This study aimed to assess the effect of RSQ on the IL-12/IL-35 balance in patients with BC.

**Subjects and methods:**

The study included 26 female patients with invasive non-metastatic BC and 20 age-matched healthy female controls. Peripheral blood mononuclear cells (PBMCs) were isolated and cultured in the presence or absence of RSQ. Levels of IL-12 and IL-35 in culture supernatants were quantified using sandwich enzyme-linked immunosorbent assay (ELISA) kits.

**Results:**

Baseline IL-35 levels were significantly higher in BC patients than in healthy controls, resulting in a significantly lower baseline IL-12/IL-35 ratio in the patient group. RSQ stimulation significantly increased IL-12 levels while significantly decreasing IL-35 levels in both groups. Consequently, the IL-12/IL-35 ratio increased significantly in both BC patients and healthy controls, with a more pronounced shift observed in the control group. Notably, RSQ-stimulated IL-35 levels remained significantly higher in BC patients compared with controls.

**Conclusion:**

These findings indicate a predominance of immunosuppressive activity in PBMCs from BC patients, largely driven by elevated IL-35 levels. Resiquimod may exert anti-tumorigenic effects in BC by shifting the IL-12/IL-35 balance toward a pro-inflammatory immune profile.

## Introduction

Breast cancer (BC) is currently the most prevalent malignancy and the fourth leading cause of cancer-related mortality worldwide [1]. In Egypt, BC has surpassed liver cancer to become the most prevalent cancer and ranks second in incidence after liver cancer [2].

Inflammation is recognized as a double-edged sword in cancer, exhibiting both pro- and anti-tumorigenic properties. In this context, cytokines—key components of the inflammatory milieu—can shift immune responses toward either tumor promotion or tumor suppression through the activation of various downstream effectors [3].

Harnessing inflammation through the therapeutic targeting of Toll-like receptors (TLRs), which serve as a bridge between innate and adaptive immune responses, has emerged as a promising strategy in cancer immunotherapy. Resiquimod (RSQ), a dual Toll-like receptor 7/8 (TLR7/8) agonist, has been widely characterized for its anti-tumor properties. These effects are mediated through multiple mechanisms, including the induction of immunostimulatory cytokine release, activation of cytotoxic T lymphocytes (CTLs) and natural killer (NK) cells, and suppression of regulatory T cells (Tregs) [4].

The well-characterized interleukin (IL)-12 cytokine family epitomizes a central axis of immune regulation by encompassing a spectrum of pro- and anti-inflammatory cytokines. While IL-12, IL-23, and IL-39 are pro-inflammatory members, IL-35 is an anti-inflammatory cytokine, and IL-27 exhibits both pro- and anti-inflammatory activities. Accordingly, different cytokines within this family exert distinct and interactive effects in relation to cancer development and tumorigenesis [5].

Interleukin-12, the archetypal member of this family, is predominantly secreted by activated antigen-presenting cells (APCs), including dendritic cells (DCs), monocytes, and macrophages, and to a lesser extent by B cells. The anti-tumorigenic role of IL-12 is mediated through multiple immunological mechanisms, including the induction of interferon (IFN)-γ secretion and T-helper 1 (Th1) differentiation, enhancement of the cytotoxic activity of cytotoxic T lymphocytes (CTLs) and natural killer (NK) cells, and reprogramming of immunosuppressive cells such as myeloid-derived suppressor cells (MDSCs) [6].

Interleukin-35 is produced primarily by regulatory T cells (Tregs) and regulatory B cells (Bregs). Elevated IL-35 levels have been positively correlated with tumor stage in several cancers, including breast and colorectal cancers. IL-35 promotes an immunosuppressive milieu that limits effective anti-tumor immune responses through two principal mechanisms: suppression of CD4⁺ and CD8⁺ T-cell effector functions and expansion of Treg-mediated immunosuppression. Consequently, IL-35 represents a potential therapeutic target that warrants further investigation [7].

As the incidence rate of BC is still increasing, identifying promising immunopathological therapeutic targets is of immense importance for the development of more efficient treatment strategies against BC. It has been demonstrated that in an experimental model of triple-negative BC (TNBC), intra-tumoral infusion of TLR 7/8 agonist decreased the development of mammary tumors, produced a T-cell-inflamed tumor microenvironment (TME)**,** and decreased metastatic dissemination to the lung [8].

Accordingly, we designed the present study to evaluate the effect of RSQ on the balance between IL-12 and IL-35, two pro- and anti-inflammatory cytokines with well-established tumor-suppressive and tumor-promoting activities, respectively, in BC patients. To the best of our knowledge, this investigation is the first to specifically examine the IL-12/IL-35 balance in human peripheral blood mononuclear cells (PBMCs) from non-metastatic BC patients following RSQ stimulation. Highlighting a previously unexplored immunoregulatory mechanism with potential implications for biomarker development and immunotherapy strategies.

## Subjects and methods

### Subjects

The present study was conducted on 46 participants who were categorized as follows:

#### Patients (breast cancer (BC) patients)

This group included 26 female patients between 31 and 72 years of age diagnosed with invasive non-metastatic BC who did not have surgery or receive prior BC treatments.

Inclusion Criteria:

- Histologically confirmed breast cancer; immunohistochemistry (IHC) was performed to determine tumor subtype.
- No prior BC treatment (surgery, chemotherapy, or radiotherapy).
- Classified as non-metastatic based on clinical evaluation and imaging (bilateral mammography, breast and axillary ultrasonography, and other imaging as indicated).

Exclusion Criteria:

- Other malignancies
- Immune-mediated diseases

#### Controls (healthy controls (HC))

This group included 20 healthy, age-matched females.

All patients were prospectively recruited from the Cancer Management and Research department and the Surgery department, Medical Research Institute, Alexandria University, Egypt, during the period between September 2022 and January 2023.

#### Ethics statement

All participants were requested to freely volunteer for the study, and informed written consents were obtained prior to their participation in the study protocol, in accordance with the ethical guidelines of the Medical Research Institute, Alexandria University (Appendix 1: Research Ethics Committee Standard Operating Procedures) [9].

### Methods

We retrieved the following data for all enrolled patients from medical records, including demographics, menstrual status, and parity, family history (FH) of cancer and FH of BC, medical history, baseline clinical characteristics, imaging including breast imaging, and pathology.

#### History taking

History retrieved from patients’ records included: date of birth, menstrual status, parity, FH of BC, and medical history for the presence of any comorbidities, such as diabetes mellitus (DM) or hypertension. The same information was obtained from participants in the control group.

#### Clinical examination

General examination: to exclude the presence of distant metastasis.

Local breast examination: to evaluate both breasts, the normal and the affected one, for the following: the breast mass site, size, and mobility; nipple retraction; skin and chest wall affection; peau d’orange and lymph node (LN) status [10].

#### Radiological investigations:

Breast imaging: Bilateral breast mammography and bilateral breast and axillary ultrasonography were performed to assess the number, site and size of tumor masses and the number and size of LNs suspicious for metastases [11]. Suspicious LNs were defined based on specific radiological criteria, e.g., size > 1 cm, cortical thickening, loss of fatty hilum, and spherical shape.

#### Pathological investigations

- Fine-Needle Aspiration Cytology (FNAC) and/or Core Needle Biopsy (CNB): to assess the type of tumor, grade, biomarker status [12].
- Histologic grade: grading was determined by the Nottingham histologic score system [13].
- Immunohistochemistry (IHC): to assess of hormone receptor status (ER, PR) and Her-2 status. Subsequently, we classified BC into: luminal A (ER + /PR + , HER2 −), luminal B (ER + /PR + HER2 +), HER2 + (ER − /PR − HER2 +), and TNBC (ER − /PR − HER2 −) [11].

#### Clinical staging

Based on the TNM classification system, the disease staging was performed in line with the seventh edition of AJCC staging system for BC [14].

#### Blood sampling

Fresh venous blood samples (5 ml) were aseptically drawn once and collected into heparinized vacutainers. (BD ® vacutainer tube).

#### Immunological procedures

##### Isolation and culture of PBMCs

Peripheral blood mononuclear cells (PBMCs) were aseptically isolated from whole venous blood samples of BC patients and healthy female groups by density gradient centrifugation over Pancoll human, density 1.077 (Pan-biotech, Germany) using sterile 15 ml Falcon tubes. The buffy coat containing PBMCs was harvested, washed with phosphate buffered saline (PBS; pH 7.4), and re-suspended in 1 ml of complete culture medium [Roswell Park Memorial Institute (RPMI) 1640 (Biowest, USA), 10% fetal bovine serum (FBS) (Biowest, USA), 1% penicillin/ streptomycin (Biowest, USA)]. Cell viability was evaluated using trypan blue exclusion test and samples having cell viability less than 95% were discarded. The concentration of viable PBMCs was calculated and adjusted at 1X10^6^/ml [15].

Isolated PBMCs were aseptically cultured in duplicates in 24-well culture plates (1 × 10^6^cells/ml/well) at 37 °C in a humidified 5% CO_2_ incubator for 48 h. either in absence or presence of 2 μg/ml R848 (Sigma, USA) [15]. As per the manufacturer’s instructions, the lyophilized endotoxin free R848 was dissolved in 1 mL of DMSO. The suspension was warmed in a 60°C water bath until it cleared. The solution was then divided into 5 μL aliquots and stored at − 20°C until further use. To prepare the R848 working solution (2μg/mL), 2 μL of TLR7/8 stock was diluted into 98 μL of RPMI 1640 complete medium at time of use.

The supernatants were then collected, aliquoted and stored at − 80℃ for later determination of IL-12 and IL-35 concentration. Repeated freeze–thaw cycles were avoided.

##### Assessment of IL-12 and IL-35

IL-12 and IL-35 were assessed in culture supernatant of isolated PBMCs using commercially available sandwich enzyme-linked immunosorbent assay (ELISA) kits according to manufacturer recommendation. The assays were done in duplicates. The results are expressed as mean of the duplicate wells. The reading variability did not exceed ± 7% of the mean. The kits used were purchased from Biospes (Chongqing, China), with the following catalogue numbers: human IL-12p70 (BZEK1850) (Range: 37.5 pg/ml–600 pg/ml; sensitivity: 3 pg/ml) and human IL-35 (BZEK1854) (Range: 37.5 pg/ml–600 pg/ml; Sensitivity: 0.3 pg/ml).

### Statistical analysis

Data were fed to the computer and analyzed using IBM SPSS software package version 20.0 (Armonk, NY: IBM Corp). Qualitative data were described using number and percent. The Shapiro-Wilk test was used to verify the normality of distribution. Quantitative data were described using range (minimum and maximum), mean ± standard deviation (SD), and median with interquartile range (IQR).Statistical comparisons between groups were performed using the Student’s *t*-test (for independent samples) or the Mann–Whitney *U* test, as appropriate. Comparisons within groups (before and after RSQ stimulation) utilized the paired *t*-test. Correlations were assessed using the Pearson correlation coefficient (*r*). Statistical significance was determined at a *p*-value ≤ 0.05, corresponding to a 95% confidence interval**.**

## Results

### Patient demographics and clinical characteristics

The study included 46 participants: 26 BC patients and 20 HC. There were no statistically significant differences between the groups in age or menopausal status (*p* > 0.05). FH of cancer or FH of BC, as well as comorbidities including DM, hypertension, and other medical conditions, were not significantly associated with BC in this cohort (*p* > 0.05 for all comparisons).

### Tumor characteristics

Among BC patients, the majority (96.2%) had invasive ductal carcinoma, with histological grade II predominating (57.7%). Tumor size ranged from > 2 cm to ≤ 5 cm in 65.4% of patients. TNM staging indicated that 57.7% of patients were stage II. Positive LN involvement was observed in 73.1% of cases. Tumor-infiltrating lymphocytes (TILs) and lymphovascular invasion (LVI) were absent in 65.4% and 61.5% of patients, respectively. Hormone receptor analysis showed that 65.4% of tumors were estrogen receptor (ER)-positive and 61.5% progesterone receptor (PR)-positive, while 88.5% of cases were HER2-negative. The most prevalent molecular subtype was luminal A (57.7%).

### Levels of pro- and anti-inflammatory cytokines

**Fig. 1:**
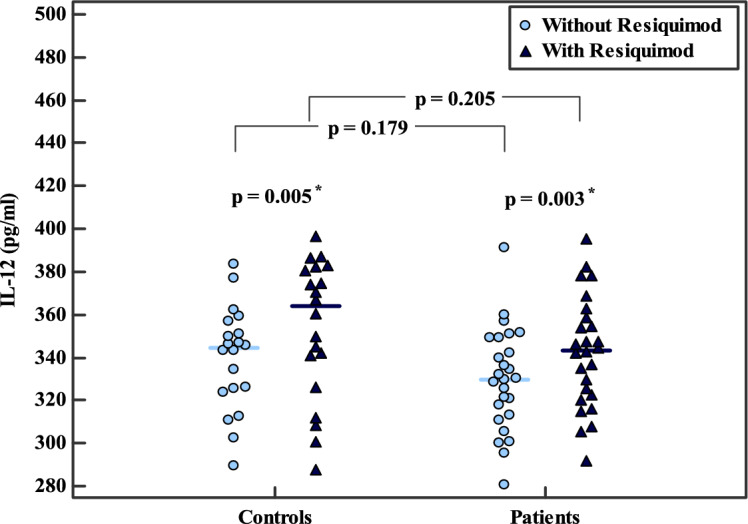
Comparison of IL-12 levels in PBMC culture supernatants before and after RSQ stimulation in HC (n = 20) and BC patients (n = 26). Statistical analysis: Student’s t-test for inter-group comparisons and paired t-test for intra-group comparisons

Baseline IL-12 levels in unstimulated PBMC supernatants did not differ significantly between healthy controls and BC patients (mean ± SD: 339.5 ± 24.0 vs. 329.8 ± 23.9 pg/ml, *p* = 0.179). Following RSQ stimulation, IL-12 levels remained comparable between the two groups (353.7 ± 32.4 vs. 342.7 ± 25.8 pg/ml, *p* = 0.205). Within-group analysis revealed a significant increase in IL-12 levels post-stimulation in both controls (*p* = 0.005) and patients (*p* = 0.003) (Table 1, Fig. 1).

**Table 1:** Comparison of IL-12 and IL-35 levels and the IL-12/IL-35 ratio in PBMC culture supernatants before and after RSQ stimulation in HC (n = 20) and BC patients (n = 26)

	HC (No. = 20)	BC (No. = 26)	Test of Sig	*p*
IL-12 (pg/ml)				
*Without RSQ*			t = 1.366	0.179
Min.–Max	289.3–383.3	280.7–391.2		
Mean ± SD	339.5 ± 24.04	329.8 ± 23.91		
Median (IQR)	344.6 (324.8–354.0)	329.9 (313.0–349.5)		
*With RSQ*			t = 1.287	0.205
Min.–Max	287.61–396.7	291.66–395.36		
Mean ± SD	353.7 ± 32.37	342.7 ± 25.79		
Median (IQR)	363.8 (333.5–381.6)	343.5 (322.6–358.4)		
t_1_(p_0_)	3.186^*^ (0.005^*^)	3.229^*^ (0.003^*^)		
Percent of increase			U = 234.0	0.565
Min.–Max	− 0.89–18.03	− 4.51–17.98		
Mean ± SD	4.14 ± 5.93	4.06 ± 6.35		
Median (IQR)	− 0.20 ( − 0.50–9.32)	1.89 ( − 0.88–8.56)		
IL-35 (pg/ml)				
*Without RSQ*			t = 3.199^*^	0.003^*^
Min.–Max	113.5–152.8	123.4–156.9		
Mean ± SD	130.5 ± 11.93	141.0 ± 10.23		
Median (IQR)	128.7 (120.1–140.7)	141.5 (134.3–150.4)		
*With RSQ*			t = 3.175^*^	0.003^*^
Min.–Max	82.94–132.8	85.46–155.2		
Mean ± SD	100.7 ± 17.22	118.8 ± 20.62		
Median (IQR)	94.95 (85.03–118.3)	121.6 (100.1–135.8)		
t_1_(p_0_)	7.877^*^ (> 0.001^*^)	6.840^*^ (> 0.001^*^)		
Percent of reduction			U = 177.00	0.066
Min.–Max	0.34–37.27	0.25–36.01		
Mean ± SD	22.71 ± 12.47	15.93 ± 12.17		
Median (IQR)	28.89 (10.18–32.58)	12.42 (5.86–25.88)		
IL-12/IL-35				
*Without RSQ*			t = 3.156*	0.003^*^
Min.–Max	1.98–3.17	2.04–2.83		
Mean ± SD	2.63 ± 0.35	2.35 ± 0.24		
Median (IQR)	2.63 (2.39–2.86)	2.35 (2.19–2.51)		
*With RSQ*			t = 3.553*	0.001^*^
Min.–Max	2.41–4.72	2.20–4.06		
Mean ± SD	3.61 ± 0.66	2.97 ± 0.55		
Median (IQR)	3.61 (3.17–4.06)	2.97 (2.60–3.34)		
t_1_(p_0_)	7.604^*^ (> 0.001^*^)	6.873* (> 0.001*)		

SD: Standard deviation, t: Student t-test, t_1_: Paired t-test, p: p value for inter-group analysis, p_0_: p value for intra-group analysis, *: Statistically significant at p ≤ 0.05. RSQ: Resiquimod, BC: breast cancer

**Fig. 2:**
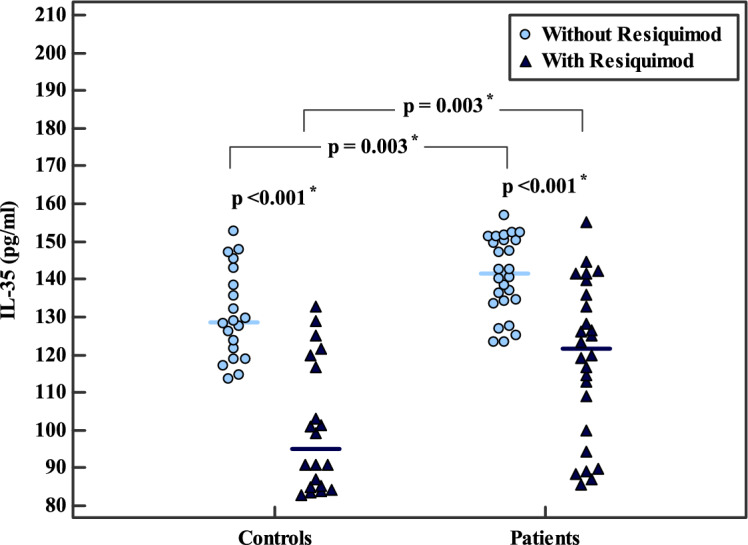
Comparison of IL-35 levels in PBMC culture supernatants before and after RSQ stimulation in HC (n = 20) and BC patients (n = 26). Statistical analysis: Student’s t-test for inter-group comparisons and paired t-test for intra-group comparisons

In contrast, IL-35 levels were significantly higher in BC patients than in controls under both unstimulated (141.0 ± 10.2 vs. 130.5 ± 11.9 pg/ml, *p* = 0.003) and RSQ-stimulated conditions (118.8 ± 20.6 vs. 100.7 ± 17.2 pg/ml, *p* = 0.003). Both groups showed significant reductions in IL-35 levels following stimulation (*p* < 0.001), with a larger percentage decrease observed in controls, approaching statistical significance (*p* = 0.066) (Table 1, Fig. 2).

**Fig. 3:**
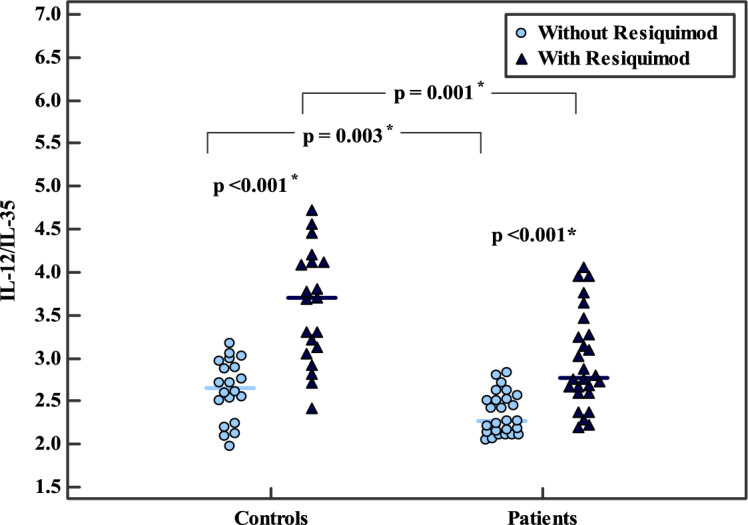
Comparison of the IL-12/IL-35 ratio in PBMC culture supernatants before and after RSQ stimulation in HC (n = 20) and BC patients (n = 26). Statistical analysis: Student’s t-test for inter-group comparisons and paired t-test for intra-group comparisons

The IL-12/IL-35 ratio was significantly lower in BC patients than in controls both before (2.35 ± 0.24 vs. 2.63 ± 0.35, *p* = 0.003) and after stimulation (2.97 ± 0.55 vs. 3.61 ± 0.66, *p* = 0.001). Stimulation significantly increased the IL-12/IL-35 ratio within both groups (*p* < 0.001), with a more pronounced increase observed in healthy controls (Table 1, Fig. 3).

### Correlation between IL-12 and IL-35 Levels

**Fig. 4:**
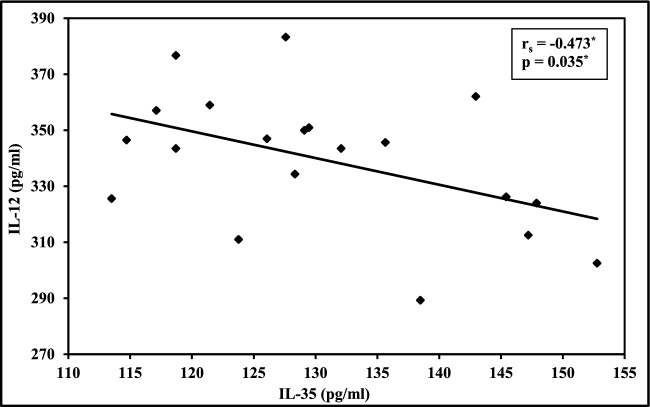
Correlation between basal levels of IL-12 and IL-35 in healthy controls (n = 20) r: Pearson coefficient, *: Statistically significant at *p* ≤ 0.05

In BC patients, there was no statistically significant correlation between IL-12 and IL-35 levels, either at baseline or following RSQ stimulation. In contrast, a significant moderate negative correlation was observed in healthy controls at baseline (r = − 0.473, *p* = 0.035), indicating an inverse relationship between IL-12 and IL-35 levels under unstimulated conditions. However, this correlation was lost after RSQ stimulation, suggesting that the cytokine balance may be independently regulated upon activation. (Fig. 4).

### Subgroup analysis for IL-12 response

IL-12 levels showed statistically significant increases following RSQ stimulation of PBMCs across several clinical and pathological subgroups (*p* < 0.05) including older patients (> 50 years), post-menopausal women, patients without FH of BC, early-stage disease, Stage II tumors, and those with ER-positive, PR-negative status. Detailed results are presented in Tables 2 and 3.

**Table 2:** Associations between IL-12 Levels (without vs. with RSQ stimulation) and clinicopathological characteristics in BC patients (n = 26)

	No	IL-12 (pg/ml)		t	*p*
		Without RSQ	With RSQ		
*Age (years)*					
≤ 50	11	320.1 ± 23.13	329.1 ± 27.50	1.413	0.188
> 50	15	336.9 ± 22.58	352.6 ± 19.93	3.049^*^	0.009^*^
*Menstrual status*					
Pre-menopausal	13	323.6 ± 21.06	335.5 ± 29.05	1.787	0.099
Post-menopausal	13	336.0 ± 25.78	349.8 ± 20.79	2.974^*^	0.012^*^
*Family history of BC*					
Yes	5	319.9 ± 15.12	332.1 ± 18.96	1.586	0.188
No	21	332.2 ± 25.27	345.19 ± 26.93	2.792^*^	0.011^*^
*Diagnosis*					
Early stage	20	329.0 ± 19.81	341.7 ± 25.12	3.003^*^	0.007^*^
Locally advanced	6	332.4 ± 36.83	346.1 ± 30.16	1.249	0.267
*Location*					
Right BC	11	338.1 ± 23.45	356.2 ± 23.89	3.325^*^	0.008^*^
Left BC	15	323.7 ± 23.10	332.8 ± 23.07	1.622	0.127
*DM*					
Yes	6	321.6 ± 21.62	344.7 ± 22.95	2.669^*^	0.044^*^
No	20	332.3 ± 24.52	342.1 ± 27.11	2.241^*^	0.037^*^
*Hypertension*					
Yes	6	332.6 ± 34.36	355.3 ± 17.29	2.750^*^	0.040^*^
No	20	329.0 ± 20.94	338.9 ± 27.04	2.227^*^	0.038^*^
*TNM Staging*					
Stage I	5	328.2 ± 20.65	341.7 ± 15.67	1.583	0.189
Stage II	15	329.3 ± 20.26	341.7 ± 28.04	2.465^*^	0.027^*^
Stage III	6	332.4 ± 36.83	346.1 ± 30.16	1.249	0.267
*Tumor site of the largest mass*					
UOQ	18	329.4 ± 20.15	345.4 ± 24.16	3.402^*^	0.003^*^
UIQ	5	324.9 ± 39.68	337.5 ± 38.62	1.178	0.304
LIQ	3	340.5 ± 17.58	335.1 ± 12.35	1.528	0.266
*Mass number*					
Single	15	327.6 ± 28.56	337.8 ± 26.40	2.352^*^	0.034^*^
Multiple	11	332.8 ± 16.42	349.3 ± 24.59	2.209	0.052
*Histopathologic Type*					
Invasive ductal carcinoma	25	331.8 ± 22.16	343.7 ± 25.75	2.961*	0.007*
Invasive mucinous carcinoma	1^#^	280.7^#^	–	**–**	**–**

SD: Standard deviation; t: Paired t-test; *p*: *p* value comparing IL-12 values (without RSQ vs with RSQ); * Statistically significant at *p* ≤ 0.05; **#** Excluded from the comparison due to small number of cases (n = 1); UOQ**:** Upper Outer Quadrant; UIQ**:** Upper Inner Quadrant; LIQ**:** Lower Inner Quadrant, RSQ: Resiquimod, BC: breast cancer

**Table 3:** Associations Between IL-12 Levels (Without and With RSQ Stimulation) and Clinicopathological Characteristics in BC Patients (n = 26) **"**continue**"**

	No	IL-12 (pg/ml)		t	*p*
		Without RSQ	With RSQ		
*Histologic grade (G)*					
GX	7	332.1 ± 17.37	353.9 ± 20.52	2.519^*^	0.045^*^
G1–G2	16	330.6 ± 27.71	340.2 ± 28.88	2.033	0.060
G3	3	320.1 ± 17.79	329.7 ± 7.10	0.711	0.551
*Mass size*					
≤ 2	9	335.2 ± 26.31	351.1 ± 20.06	2.110	0.068
> 2 – ≤ 5	17	327.0 ± 22.85	338.2 ± 27.88	2.376^*^	0.030^*^
*Lymph nodes (LNs)*					
Positive	19	331.5 ± 25.98	345.9 ± 27.55	2.954^*^	0.008^*^
Negative	7	325.3 ± 18.04	333.8 ± 19.20	1.256	0.256
*Lymphocytic infiltration (LI)*					
Positive	9	330.8 ± 13.97	336.6 ± 14.47	1.246	0.248
Negative	17	329.3 ± 28.19	345.9 ± 30.04	3.050^*^	0.008^*^
*Lymphovascular invasion (LVI)*					
Positive	10	328.1 ± 26.50	343.0 ± 22.78	2.926^*^	0.017^*^
Negative	16	330.9 ± 22.98	342.5 ± 28.24	2.019	0.062
*T staging*					
cT1	8	328.2 ± 16.91	347.2 ± 17.34	2.432^*^	0.045^*^
cT2	15	326.07 ± 23.54	338.7 ± 28.82	2.388^*^	0.032^*^
cT4	3	352.9 ± 36.79	350.8 ± 33.89	0.519	0.655
cT1–cT2	23	326.8 ± 21.08	341.6 ± 25.33	3.425^*^	0.002^*^
cT4	3	352.9 ± 36.79	350.8 ± 33.89	0.519	0.655
*N staging*					
cN0	6	327.73 ± 18.50	338.5 ± 16.03	1.440	0.210
cN1	15	328.8 ± 20.46	341.2 ± 28.47	2.467^*^	0.027^*^
cN3	5	335.3 ± 40.40	352.3 ± 29.14	1.326	0.255
*Estrogen receptor (ER)*					
Positive	17	335.5 ± 25.39	346.3 ± 24.48	2.282^*^	0.037^*^
Negative	9	319.0 ± 17.24	335.8 ± 28.29	2.245	0.055
*Progesterone receptor (PR)*					
Positive	16	333.9 ± 26.08	344.5 ± 23.09	1.972	0.067
Negative	10	323.3 ± 19.40	339.8 ± 30.74	2.767^*^	0.022^*^
*HER 2 Neu*					
Positive	3	347.2 ± 15.37	367.0 ± 25.18	1.179	0.360
Negative	23	327.5 ± 24.11	339.5 ± 24.64	2.924^*^	0.008^*^
*Molecular subtype*					
Luminal A	15	332.2 ± 26.03	344.3 ± 23.88	2.208^*^	0.044^*^
Luminal B	3	347.2 ± 15.37	367.0 ± 25.18	1.179	0.36
Triple negative	8	318.8 ± 18.42	330.6 ± 25.04	1.883	0.102

SD: Standard deviation; t: Paired t-test; *p*: *p* value *: Statistically significant at *p* ≤ 0.05, RSQ: Resiquimod, BC: breast cancer

### Subgroup analysis for IL-35 response

IL-35 levels significantly decreased following RSQ stimulation of PBMCs across the majority of clinical and pathological subgroups (*p* < 0.05), including both age groups, pre- and post-menopausal patients, early-stage and locally advanced disease, and most stages and hormone receptor subtypes. Detailed results are presented in Tables 4 and 5.

**Table 4:** Associations between IL-35 levels (without and with RSQ stimulation) and clinicopathological characteristics in BC patients (n = 26)

	No	IL-35 (pg/ml)		t	*p*
		Without RSQ	With RSQ		
*Age (years)*					
≤ 50	11	138.0 ± 11.14	107.3 ± 19.17	6.909^*^	< 0.001^*^
> 50	15	143.2 ± 9.27	127.2 ± 17.82	4.028^*^	0.001^*^
*Menstrual status*					
Pre-menopausal	13	140.7 ± 9.58	114.7 ± 21.84	5.354^*^	< 0.001^*^
Post-menopausal	13	141.3 ± 11.22	122.9 ± 19.32	4.347^*^	0.001^*^
*Family history of BC*					
Yes	5	147.3 ± 5.33	132.7 ± 11.64	2.199	0.093
No	21	139.5 ± 10.62	115.5 ± 21.09	6.571^*^	< 0.001^*^
*Diagnosis*					
Early stage	20	142.21 ± 9.62	119.7 ± 20.55	5.933^*^	< 0.001^*^
Locally advanced	6	137.0 ± 12.08	115.7 ± 22.51	3.132^*^	0.026^*^
*Location*					
Right BC	11	141. 5 ± 10.52	126.2 ± 18.90	3.367^*^	0.007^*^
Left BC	15	140.7 ± 10.36	113.4 ± 20.72	6.535^*^	< 0.001^*^
*DM*					
Yes	6	138.9 ± 7.57	124.2 ± 19.19	2.105	0.089
No	20	141.6 ± 10.99	117.2 ± 21.23	6.777^*^	< 0.001^*^
*Hypertension*					
Yes	6	140.7 ± 8.69	122.0 ± 20.05	2.376	0.063
No	20	141.1 ± 10.85	117.8 ± 21.20	6.479^*^	< 0.001^*^
*TNM Staging*					
Stage I	5	145.0 ± 7.97	119.0 ± 22.19	3.249^*^	0.031^*^
Stage II	15	141.3 ± 10.18	120.0 ± 20.80	4.827^*^	< 0.001^*^
Stage III	6	137.0 ± 12.08	115.7 ± 22.51	3.132^*^	0.026^*^
*Tumor site of the largest mass*					
UOQ	18	139.2 ± 11.05	116.1 ± 21.43	5.611^*^	< 0.001^*^
UIQ	5	141.58 ± 6.62	123.1 ± 21.7	2.45	0.07
LIQ	3	151.0 ± 1.43	128.1 ± 15.27	2.712	0.113
*Mass number*					
Single	15	138.5 ± 10.38	113.2 ± 23.0	5.341^*^	< 0.001^*^
Multiple	11	144.4 ± 9.43	126.5 ± 14.48	4.485^*^	0.001^*^
*Histopathologic Type*					
Invasive ductal carcinoma (IDC)	25	141.7 ± 9.77	120.1 ± 20.0	6.504*	< 0.00*
Invasive mucinous carcinoma (IMC)	1^#^	123.4^#^	86.93^#^	**–**	**–**

SD: Standard deviation; t: Paired t-test; *p*: *p* value comparing IL-12 values (without RSQ vs with RSQ); * statistically significant at *p* ≤ 0.05; **#** Excluded from the comparison due to small number of cases (n = 1); UOQ**:** Upper Outer Quadrant; UIQ**:** Upper Inner Quadrant; LIQ**:** Lower Inner Quadrant, RSQ: Resiquimod

**Table 5:** Associations Between IL-35 Levels (Without and With RSQ Stimulation) and Clinicopathological Characteristics in BC Patients (n = 26)

	No	IL-35 (pg/ml)		t	*p*
		Without RSQ	With RSQ		
*Histologic grade (G)*					
GX	7	143.2 ± 10.05	118.7 ± 17.38	3.788^*^	0.009^*^
G1–G2	16	140.4 ± 10.16	115.7 ± 22.28	6.145^*^	< 0.001^*^
G3	3	139.1 ± 14.28	135.6 ± 13.69	1.942	0.192
*Mass size*					
≤ 2	9	142.0 ± 8.79	123.6 ± 17.36	3.471^*^	0.008^*^
> 2 – ≤ 5	17	140.5 ± 11.13	116.3 ± 22.23	5.870^*^	< 0.001^*^
*Lymph nodes (LNs)*					
Positive	19	140.15 ± 10.43	119.0 ± 19.82	5.557^*^	< 0.001^*^
Negative	7	143.3 ± 10.04	118.3 ± 24.37	3.811^*^	0.009^*^
*Lymphocytic infiltration (LI)*					
Positive	9	143.9 ± 11.77	128.4 ± 13.11	3.412^*^	0.009^*^
Negative	17	139.5 ± 9.33	113.7 ± 22.34	6.170^*^	< 0.001^*^
*Lymphovascular invasion (LVI)*					
Positive	10	144.5 ± 8.99	134.7 ± 11.58	4.251^*^	0.002^*^
Negative	16	138.8 ± 10.61	108.9 ± 18.88	7.432^*^	< 0.001^*^
*T staging*					
cT1	8	142.5 ± 9.28	123.6 ± 18.56	3.145^*^	0.016^*^
cT2	15	140.1 ± 11.54	116.9 ± 22.25	5.260^*^	< 0.001^*^
cT4	3	141.6 ± 7.66	115.4 ± 22.58	2.613	0.121
cT1–cT2	23	140.9 ± 10.65	119.3 ± 20.86	6.199^*^	< 0.001^*^
cT4	3	141.6 ± 7.66	115.4 ± 22.58	2.613	0.121
*N staging*					
cN0	6	146.1 ± 7.57	123.3 ± 22.45	3.120^*^	0.026^*^
cN1	15	141.2 ± 10.12	119.2 ± 19.99	5.127^*^	< 0.001^*^
cN3	5	134.3 ± 11.35	112.3 ± 23.36	2.663	0.056
*Estrogen receptor (ER)*					
Positive	17	138.2 ± 10.40	113.3 ± 16.54	6.481^*^	< 0.001^*^
Negative	9	146.2 ± 7.98	129.2 ± 24.39	2.919^*^	0.019^*^
*Progesterone receptor (PR)*					
Positive	16	138.67 ± 10.87	115.0 ± 17.77	5.700^*^	< 0.001^*^
Negative	10	144.8 ± 8.26	125.0 ± 24.22	3.682^*^	0.005^*^
*HER 2 Neu*					
Positive	3	143.8 ± 8.25	114.3 ± 14.02	5.880^*^	0.028^*^
Negative	23	140.7 ± 10.56	119.4 ± 21.50	5.922^*^	< 0.001^*^
*Molecular subtype*					
Luminal A	15	137.0 ± 10.85	115.0 ± 18.40	5.249^*^	< 0.001^*^
Luminal B	3	143.8 ± 8.25	114.3 ± 14.02	5.880^*^	0.028^*^
Triple negative	8	145.7 ± 8.37	127.7 ± 25.60	2.773^*^	0.028^*^

SD: Standard deviation; t: Paired t-test; *p*: *p* value comparing IL-135 values (without RSQ vs with RSQ); *: Statistically significant at *p* ≤ 0.05, RSQ: Resiquimod

### Association between IL-12/IL-35 ratio and clinicopathological features in BC patients

Given the significant increase in the IL-12/IL-35 ratio following RSQ stimulation in BC patients, further analysis was performed to examine its relationship with clinicopathological features. Overall, no significant associations were observed between the IL-12/IL-35 ratio and most clinical parameters. However, the ratio was significantly higher in patients who were negative for LI and LVI compared to those who were positive (*p* < 0.05). These results suggest that a higher IL-12/IL-35 ratio, reflecting a more pro-inflammatory immune profile, may be associated with the absence of LI and LVI, which are established markers of tumor aggressiveness (Table 6).

**Table 6:** Associations between IL-12/IL-35 ratio and clinicopathological features in BC patients following RSQ stimulation (n = 26)

	No	IL-12/IL-35	Test of significance	*p*
		Mean ± *SD*		
*Age (years)*				0.156
≤ 50	11	3.15 ± 0.59	t =	
> 50	15	2.83 ± 0.51	1.464	
*TNM Staging*				0.854
Stage I	5	2.97 ± 0.68	F =	
Stage II	15	2.92 ± 0.53	0.159	
Stage III	6	3.08 ± 0.61		
*Mass size*				0.648
≤ 2	9	2.90 ± 0.50	t =	
> 2 – ≤ 5	17	3.01 ± 0.59	0.463	
*Lymph nodes (LNs)*				0.849
Positive	19	2.98 ± 0.53	t =	
Negative	7	2.93 ± 0.66	0.193	
*Histologic grade (n = 19)*				0.119
GI – GII		3.04 ± 0.60	t =	
GIII	16	2.45 ± 0.27	1.643	
*Lymphocytic infiltration (LI)*				0.008^*^
Positive	9	2.64 ± 0.29	**t = **	
Negative	17	3.14 ± 0.59	2.884^*^	
*Lymphovascular invasion (LVI)*				< 0.001^*^
Positive	10	2.57 ± 0.29	**t = **	
Negative	16	3.22 ± 0.54	4.034^*^	
*Estrogen receptor (ER)*				0.057
Positive	17	3.12 ± 0.50	t =	
Negative	9	2.69 ± 0.58	1.999	
*Progesterone receptor (PR)*				*0.308*
Positive	16	3.06 ± 0.47	t =	
Negative	10	2.83 ± 0.67	1.041	
*HER2*				0.343
Positive	3	3.26 ± 0.61	t =	
Negative	23	2.93 ± 0.55	0.968	
*Molecular subtype*				0.198
Luminal A		3.06 ± 0.48	F =	
Luminal B	15	3.26 ± 0.61	1.737	
Triple negative	3	2.69 ± 0.62		

SD: Standard deviation, t: Student t-test, F: F for One way ANOVA test, *p*: *p* value for relation between IL-12/IL-35 and different parameters, *: Statistically significant at *p* ≤ 0.05. RSQ: Resiquimod

## Discussion

Inflammation plays a dual role in cancer, including breast cancer (BC), where it can either suppress or promote tumor development [16–18]. Several cancer therapies exploit inflammatory pathways to stimulate antitumor immune responses via cytokine modulation [19, 20]. In this context, our study evaluated the immunological effects of the TLR7/8 agonist RSQ, focusing on IL-12 and IL-35, two cytokines representing opposing immune pathways: IL-12 as a pro-inflammatory driver of antitumor immunity, and IL-35 as an immunosuppressive mediator of tumor immune evasion. Together, these cytokines provide a meaningful measure of immune balance in BC.

The demographic and clinicopathological characteristics of our cohort are consistent with regional trends. The mean age of patients was 50.85 years, closely matching pooled Egyptian data (50.46 years), with 65% being premenopausal [21]. Disease presentation was predominantly stage II, with most tumors sized > 2– ≤ 5 cm and 73.1% showing LN involvement, reflecting advanced disease at diagnosis, a pattern commonly observed in Egypt but differing from other populations such as China [21–23]. Histologically, the majority of tumors were invasive ductal carcinoma of no special type (IDC-NST), with grade II being most prevalent (57.7%) [19, 24, 25]. TILs and LVI were present in 35% and 39% of cases, respectively, values consistent with reported variability due to interobserver differences [22, 26, 27].

Hormone receptor expression followed expected patterns, with 65.4% ER-positive, 61.5% PR-positive, and 11.5% HER2-positive tumors [22]. In the absence of Ki-67 data, IHC-based classification identified luminal A as the predominant molecular subtype (57.7%), followed by triple-negative (30.8%) and luminal B (11.5%), aligning with earlier studies [23]. These detailed clinicopathological data provide essential context for interpreting the immunomodulatory effects of RSQ on IL-12 and IL-35 in BC patients.

IL-12, a critical Th1 cytokine, exerts well-established antitumor effects through activation of cytotoxic T lymphocytes (CTLs), natural killer (NK) cells, and interferon-gamma (IFN-γ) production [28–32]. In our study, IL-12 expression was significantly upregulated following RSQ stimulation in both BC patients and healthy controls, confirming that innate immune responsiveness is preserved in BC despite an overall immunosuppressive environment [29–32]. This finding is particularly relevant given prior evidence of IL-12/STAT4 pathway impairment in BC, often mediated by IL-10 [33].

Conversely, IL-35, produced primarily by regulatory T (Treg) and B (Breg) cells, was significantly elevated in BC patients at baseline and post-RSQ. IL-35 is known to suppress effector T cell function, enhance Treg activity, and promote immune evasion, contributing to poor prognosis and disease progression [34–38]. Although RSQ reduced IL-35 levels in both groups, the reduction was less pronounced in BC patients, indicating persistent immune suppression even under pro-inflammatory stimulation.

The IL-12/IL-35 ratio provides a meaningful metric of the immune milieu. In our cohort, this ratio was significantly lower in BC patients at baseline and after RSQ stimulation, reflecting a dominant immunosuppressive profile. RSQ increased the ratio in both groups, with a stronger response in healthy controls, highlighting relative immune resistance in BC patients [31]. The absence of correlation between IL-12 and IL-35 post-stimulation further suggests a dysregulated cytokine network in BC.

The tumor microenvironment (TME) in BC is often enriched with IL-35–producing Tregs, particularly in aggressive subtypes, correlating with poor clinical outcomes and negative hormonal status [35, 36]. These factors may limit the efficacy of monotherapies like RSQ, suggesting the need for combinatorial approaches targeting multiple immune checkpoints and cytokines [39–43].

Clinically, RSQ-induced IL-12 upregulation and IL-35 reduction were observed across a broad range of patient subgroups. IL-12 responses were particularly notable in patients over 50, postmenopausal women, and those without a FH of BC. Tumors that were early-stage, stage II, right-sided, or located in the upper outer quadrant exhibited significant IL-12 induction, potentially reflecting enhanced lymphatic drainage and immune surveillance in these anatomical areas [44]. Interestingly, patients with metabolic comorbidities such as DM and hypertension also showed significant IL-12 responses, suggesting that RSQ may partially overcome systemic inflammation–associated immune suppression.

Furthermore, IL-12 upregulation was evident in tumors with positive LNs and absent TILs, highlighting RSQ’s potential to activate “cold” tumors [45]. Enhanced IL-12 expression in luminal A and ER-positive subtypes, coupled with improvements in the IL-12/IL-35 ratio, underscores RSQ’s therapeutic relevance even in less immunogenic or less aggressive BC forms [46, 47]. These findings support the broader immunomodulatory potential of RSQ across diverse clinical and pathological settings.

Similarly, IL-35 levels were significantly reduced across many subgroups, including early-stage disease, tumors ≤ 5 cm, lower histological grades, and both LN-positive and -negative patients. The reduction was more evident in patients without a FH, hinting at immunological differences between hereditary and sporadic cases. IL-35 suppression across hormone receptor subtypes, including TNBC and HER2-positive cases, highlights RSQ’s broad applicability [7, 48].

IL-12 and IL-35 were selected because they represent two functionally opposing and mechanistically relevant cytokine axes within BC immunology and TLR7/8 signaling. IL-12 is a prototypical pro-inflammatory, Th1-driving cytokine produced primarily by dendritic cells (DCs) following TLR7/8 activation and is essential for effective antitumor immunity through activation of cytotoxic T lymphocytes, natural killer cells, and IFN-γ production [42, 45]. In contrast, IL-35 is a key immunosuppressive cytokine predominantly produced by regulatory T cells, where it contributes to immune tolerance, inhibition of effector T-cell responses, and tumor immune evasion in BC [7, 48].

TLR7/8 agonists such as RSQ stimulate DC maturation and cytokine release, favoring IL-12–mediated immune activation while modulating IL-35–producing regulatory T cells, thereby shifting the immune balance toward antitumor responses [42, 43, 49]. Assessing IL-12 and IL-35 thus provides a meaningful readout of how TLR7/8 signaling influences the balance between pro- and anti-inflammatory pathways in BC, offering mechanistic insight relevant to immunotherapeutic strategies.

Although this study focused on IL-12 and IL-35, TLR7/8 activation also induces a broader cytokine network—including IL-6, TNF-α, IFN-α, and IFN-γ—that amplifies DC activation and effector T-cell function [42, 43]. While these were not directly measured here, their known induction provides important context for interpreting the observed shift in the IL-12/IL-35 axis.

Importantly, TLR agonists such as imiquimod have been shown to modulate regulatory T-cell function and diminish their suppressive influence within the tumor microenvironment, thereby tipping the immune balance toward antitumor responses in both mouse models and human tumors. Together, these effects illustrate how TLR agonists can strengthen antitumor immunity by promoting effector T-cell activity while functionally restraining Tregs, highlighting their potential in combination immunotherapy approaches. [47, 50].

Together, these findings support the immunomodulatory role of RSQ, reflected by the dual increase in IL-12 and decrease in IL-35, especially in patients with less aggressive disease. This shift suggests a potential therapeutic window where RSQ could enhance immune responses or synergize with other treatments such as checkpoint inhibitors or IL-35 blockade [51, 52].

Subgroup analysis also revealed that IL-12/IL-35 ratio improvements post-RSQ were more significant in patients lacking LI and LVI, further supporting its utility in less aggressive tumors. However, this is nuanced by conflicting evidence where increased TILs or LI has been associated with poorer survival in specific BC subtypes, underscoring the heterogeneity of the immune landscape in BC [53].

A key consideration in interpreting our findings is that the immunomodulatory effects observed following RSQ stimulation may reflect generalized TLR7/8 pathway activation rather than compound-specific activity. RSQ was selected for its well-characterized dual TLR7/8 agonism and translational relevance, but the absence of experiments using TLR7- or TLR8-selective agonists limits the ability to dissect receptor-specific contributions to IL-12 induction and IL-35 suppression. Therefore, the cytokine shifts reported here should be viewed as downstream effects of pharmacologic TLR7/8 engagement. Future studies employing receptor-specific agonists, antagonists, or genetic approaches are warranted to determine whether RSQ provides immunological advantages beyond general TLR7/8 activation and to clarify its optimal role in BC immunotherapy.

## Limitations

This study has several limitations. The relatively small cohort size limits statistical power for subgroup analyses, and the single post-stimulation time point restricts insight into dynamic cytokine responses. PBMCs represent a heterogeneous mixture of immune cells: IL-12 is primarily produced by monocytes and dendritic cells, whereas IL-35 is mainly secreted by regulatory T and B cells. Consequently, the exact cellular sources of IL-12 and IL-35 could not be determined, and the results reflect overall cytokine balance rather than cell-type–specific effects. Future research incorporating longitudinal sampling, larger and more diverse cohorts, and cell-specific analyses is necessary to fully elucidate the immunological effects of RSQ and optimize its therapeutic potential in breast cancer (BC).

## Conclusions

The findings of this study highlight the critical balance between IL-12 and IL-35 in PBMCs of breast cancer (BC) patients. Our results indicate a predominantly immunosuppressive profile in these patients, largely driven by elevated IL-35. Stimulation of PBMCs with RSQ shifted this cytokine balance toward a more immune-stimulatory state, primarily marked by a significant reduction in IL-35 levels. This suggests a potential mechanism by which RSQ enhances effector immune responses.

## Recommendations

Based on the current findings, future research should focus on further elucidating the immunomodulatory role of RSQ in breast cancer (BC). Specifically, it is important to investigate RSQ’s effect on IL-12 and IL-35-producing immune cells within tumor, potentially using flow cytometry to determine cell-type–specific responses and compare them with those observed in PBMCs Larger cohort studies are needed to assess RSQ’s influence across various disease phenotypes and progression stages. Examining cytokine release kinetics from PBMCs at multiple time points following RSQ exposure could further clarify its temporal effects. Finally, the development and evaluation of innovative RSQ formulations specifically designed for BC patients’ immune cells may enhance therapeutic efficacy while minimizing adverse effects. Assessing cell viability in culture is another relevant parameter that could be incorporated in future studies.

## Data Availability

All data generated, including those that support the findings of the current study, are available from the corresponding author upon reasonable request.
